# Conducting Causal Analysis by Means of Approximating Probabilistic Truths

**DOI:** 10.3390/e24010092

**Published:** 2022-01-06

**Authors:** Bo Pieter Johannes Andrée

**Affiliations:** 1 Analytics and Tool Unit, Development Economics Data Group, World Bank, 1818 H St NW, Washington, DC 20433, USA; bandree@worldbank.org or b.p.j.andree@vu.nl; 2 Department of Spatial Economics, School of Business and Economics, VU University Amsterdam, De Boelelaan 1105, 1081 HV Amsterdam, The Netherlands

**Keywords:** causality, Bitcoin, inflation, yield spreads, approximation theory, Hellinger distance, Kullback–Leibler divergence, correct specification, misspecified models

## Abstract

**Simple Summary:**

The current paper develops a probabilistic theory of causation and suggests practical routines for conducting causal inference applicable to new machine learning methods that have, so far, remained relatively underutilized in this context.

**Abstract:**

The current paper develops a probabilistic theory of causation using measure-theoretical concepts and suggests practical routines for conducting causal inference. The theory is applicable to both linear and high-dimensional nonlinear models. An example is provided using random forest regressions and daily data on yield spreads. The application tests how uncertainty in short- and long-term inflation expectations interacts with spreads in the daily Bitcoin price. The results are contrasted with those obtained by standard linear Granger causality tests. It is shown that the suggested measure-theoretic approaches do not only lead to better predictive models, but also to more plausible parsimonious descriptions of possible causal flows. The paper concludes that researchers interested in causal analysis should be more aspirational in terms of developing predictive capabilities, even if the interest is in inference and not in prediction per se. The theory developed in the paper provides practitioners guidance for developing causal models using new machine learning methods that have, so far, remained relatively underutilized in this context.

## 1. Introduction

Philosophers have debated at length whether causality is a subject that should be treated probabilistically or deterministically. This resulted in the development of different inferential systems and views on reality. Pure logic dealt with inferences about deterministic truths [1,2]. Probabilistic reasoning has been developed to allow for uncertainty in inferences about deterministic truths [3,4], to make inferences about probabilistic truths [5,6], or to imply the existence of associated deterministic truths [7,8,9,10,11]. Probabilistic theories about causality were developed throughout the 20th century, with notable contributions by Reichenbach, Good, and Suppe [12]. At the same time, however, the classical model of physics maintained its position as a role model for other sciences, which led researchers, including those concerned with human behavior and economic systems, to reject ideas about probabilistic causation, opting, often, to reason probabilistically about deterministic truths.

In modern physics, the standard equations of quantum mechanics suggest that reality is, in fact, better described by probability laws [13]. The outcome of the Bohr–Einstein debates settled on the assertion that these probability laws are a result of a real indeterminacy and that reality itself is probabilistic (One may also argue that this is simply a correct *exposition of the theory* and not necessarily of the physical world, as more complete theories may yet be discovered). Ref. [14] provides an alternative interpretation of quantum physics in which the probability laws are statistical results of the development of completely determined, but hidden, variables. At a macroscopic level, deterministic laws and contingencies induce associated probabilistic laws (Contingencies is a term used by Ref. [14] to refer to independent factors that may exist outside the scope of what is treated by the laws under consideration, and which do not follow necessarily from anything that may be specified under the context of these laws). In particular, by broadening the context of the processes under consideration, new laws that govern some of the contingencies can be found. This inevitably leads to new contingencies: a process that repeats indefinitely. For this reason, any theory about reality that embraces either of deterministic law or chance, to the exclusion of the other, is inherently incomplete. Regardless of one’s position on real indeterminism, it holds, according to this logic, that any natural process that arises deterministically must also satisfy statistical laws that are more general, and so any complete theory about interesting real-world phenomena must be probabilistic.

In a probabilistic view of reality cause and consequence are related by probability laws rather than laws of logical truths. A theory about probabilistic causality can, therefore, be stated in terms of the properties of the *true* measure that describes a process stochastically. The theory of causation developed here is that a causal relationship exists if there exists a *true* probability measure that produces a non-empty stochastic sequence that describes the directly caused effects from perturbations in one variable in terms of the responses in another. The paper shows that ideas about causality, including the direction, statistical significance, and economic relevance of effects, may be tested by formulating a statistical model that correctly describes observed data, and evaluating its dynamic properties. In practice, this means that the inference is conducted with a best approximation of the true probability measure. It is the position of the paper that in order to demonstrate that causality runs from a potential causal variable to the target variable, one requires developing the best approximation of the true probability measure using the potential causal variable and a best approximation of the true probability measure without the potential causal variable. The analysis should then (1) conclude whether the first modeled measure is closer to the true measure, and (2) test that the two modeled measures are not equivalent. Practical routines to do so shall be discussed and an example is provided using random forest (RF) regressions and daily data on yield spreads. The application tests how uncertainty around short- and long-term inflation expectations interact with spreads in the daily Bitcoin price, a digital asset with a predetermined finite supply that has been characterized as a new potential inflation hedge. The results are contrasted with those obtained with standard linear Granger causality tests. It is shown that the suggested approaches do not only lead to better predictive models, but also to more plausible parsimonious descriptions of possible causal flows.

The focus on approximating a correct stochastic representation of the DGP (data generating process) as a means of learning about true causal linkages is different from the approaches that try to simulate laboratory conditions by testing for statistical differences in control groups, such as described by [15,16]. The focus on obtaining a correct functional representation of the data is also different from attributing the presence of causal relationships directly to the values of parameters representing averages in treatment groups, see for instance [17,18,19] on this approach. Placing emphasis on the need for accurate statistical models for the full data distribution when conducting causal analysis introduces an obvious weakness: it is generally accepted that all empirical models will be mis-specified to a certain degree and that empirical models are likely never correctly specified. The *true* process, after all, is unknown in practice. This is the reason to conduct analyses in the first place. The aim to develop correct models can therefore be seen as an idealistic idea that is difficult to put into practice. However, it is still valuable to understand the role of the correct-specification assumption in causal analysis. It is commonly taught that mis-specification leads to residual dependencies that violate the assumptions made by general central limit theorems needed to obtain correct standard errors, see for example chapter 2 in [20]. However, more general estimation theory for dependent processes, as those developed and discussed for instance by [21,22,23,24,25], may help correct standard error estimation but do not remedy the issue that the structural response of the model is incorrect [26]. These are theories to correct the variance estimator when the underlying model is wrong, and do not address the issue that the structural response of the model does not correctly describe the data.

The paper builds on contributions of others in the following lines of research. The views on causality developed in the paper are related to the information theoretic view on testing causal theories, as discussed by [27,28,29,30], which, as here, emphasizes model parsimony. The line of reasoning is inspired by the work of [31,32], who emphasized the importance of a probabilistic formulation of economic theories and warned against the use of statistical methods without any reference to a stochastic process. The paper also emphasizes the importance of the overall model response, and, thus, on focusing on system behavior, rather than on isolated parameters that make no reference to a wider economic system. This has previously been advocated by [33]. The main result of the paper is that convincing statements about partial causal linkages must be underpinned by an accurate model of broader reality, even if the interest is in inference and not prediction per se. In order to do so, researchers must, as shall be discussed, pay due attention to distinguishing between direct causal impacts and system memory and take note of developments in the field of predictive modeling.

The plan of the paper is as follows. Section 2 develops definitions for probabilistic causality in terms of true probability measures using a flexible type of dynamical system that covers many processes observed in economics, physics, finance, and related fields of study. Section 3 discusses approximating this true probability measure as an act of minimizing divergence between the modeled probability measure and the true probability measure, while Section 4 forges the link between statistical divergence and distance. This draws the connections between distance-minimization and the use of maximum likelihood criteria. Section 5 provides practical considerations and applies the theory. Finally, Section 6 concludes. Proofs are provided in the Appendix A.

## 2. Causality in Terms of True Probability Measures

Notation will be as follows.

**Notation** **1.** *N, Z and R, respectively denote the sets of natural, integer, and real numbers. If A is a set, B(A) denotes the Borel-σ algebra over A, and $\times_{t=1}^{t=T}A$, alternatively denoted as $A_{T}$, is the Cartesian product of T copies of A. Definitional equivalence is denoted :=, which is to be distinguished from ≡ denoting equivalence, for example in the functional sense. For two maps, f and g, their composition arises from their point-wise application and is denoted f ○ g:=f(g) and $f^{-1}$ is the inverse function of f. The tensor product is denoted ⊗. The notation μ≪ν is used to indicate that μ is absolutely continuous with respect to ν, i.e., if μ and ν are two measures on the same measurable space (X,A), μ is absolutely continuous with respect to ν if μ(A)=0 for every set A for which ν(A)=0, or, as an example, if ν is the counting measure on [0,1] and μ is the Lebesgue measure, then μ≪ν. It is also said that ν is* dominating * μ when μ≪ν, see for instance ([34] p. 574). Finally, the empty set* ∅ *is also used in the context of an empty sequence, which sometimes would be notated as () in the literature.*

Directional causality is interesting when at least two sequences are considered. Specifically, when the focus is on a *T*-period sequence ${\left\{x_{t}\left(\omega \right)\right\}}_{t=1}^{T}$, that is a subset of the realized path of the $n_{x}$-variate stochastic sequence $x\left(\omega \right):={\left\{x_{t}\left(\omega \right)\right\}}_{t\in Z}$ for events in the event space ω∈Ω. (That is, $x_{t}\left(\omega \right)\in X\subseteq R^{n_{x}}\;\forall \;\left(\omega ,t\right)\in \Omega \times Z$. The random sequence x(ω) is a Borel-σ $F/B(X_{\infty })$-measurable map $x:\Omega \to X_{\infty }\subseteq R_{\infty }^{n_{x}}$. In this, $R_{\infty }^{n_{x}}:=\times_{t=-\infty }^{t=\infty }R^{n_{x}}$ denotes the Cartesian product of infinite copies of $R^{n_{x}}$ and $X_{\infty }=\times_{t=-\infty }^{t=\infty }X$ with $B\left(X_{\infty }\right):=B\left(R_{\infty }^{n_{x}}\right)\cap X_{\infty }$, and $B(R_{\infty }^{n_{x}})$ denotes the Borel-σ algebra on the finite dimensional cylinder set of $R_{\infty }^{n_{x}}$, see Theorem 10.1 of [35], p. 159). As always, the complete probability space of interest is described by a triplet (Ω,F,P), with F as the σ-field defined on the event space. P is used here informally as a placeholder for a collection of probability measures, as we shall introduce the exact probability measures of interest shortly.

If x is considered as a univariate sequence independent from causal drivers, then for every event ω∈Ω, the stochastic sequence $x_{t}\left(\omega \right)$ would live on the probability space $\left(X_{\infty },B\left(X_{\infty }\right),P^{x}\right)$ where $P^{x}$ assigns probability to all elements of $B(X_{\infty })$. In a similar fashion, one can consider ${\left\{y_{t}\left(\omega \right)\right\}}_{t=1}^{T}$ as the subset of the realized path of the $n_{y}$-variate stochastic sequence $y\left(\omega \right):={\left\{y_{t}\left(\omega \right)\right\}}_{t\in Z}$ indexed by identical *t* for events ω∈Ω (i.e., $y_{t}\left(\omega \right)\in Y\subseteq R^{n_{y}}\;\forall \;\left(\omega ,t\right)\in \Omega \times Z$ and the random sequence y(ω) is a Borel-σ $F/B(Y_{\infty })$-measurable map $y:\Omega \to Y_{\infty }\subseteq R_{\infty }^{n_{y}}$.) If y would live similarly isolated from outside influence, then for every ω∈Ω, the stochastic sequence $y_{t}\left(\omega \right)$ would operate on a space $\left(Y_{\infty },B\left(Y_{\infty }\right),P^{y}\right)$ where $P^{y}$ assigns probability to all the elements of $B(Y_{\infty })$. We have a system of two unrelated sequences (This naturally covers to most common auto-regression case, only stated for $y_{t}$ here, $y_{t}=f^{yy}\left(y_{t-1}\right)+\varepsilon_{t}$, where $\varepsilon_{t}$ is unobserved. The linear auto-regression case is obtained when $f^{yy}$ is a scaled identity function.):

(1) $$\begin{array}{c} x:=\{x_{t}=f^{xx}\left(x_{t-1}\right),t\in Z\}\\ y:=\{y_{t}=f^{yy}\left(y_{t-1}\right),t\in Z\} \end{array}.$$

As we shall see, an important aspect of causal analysis is to rule out that the observed data is not generated by Equation (1). As such, it is important to comment on a number of properties. First, in this system of equations, the functions $f^{xx}$ and $f^{yy}$ are intentionally not indexed by *t*. This does not imply that these functions cannot posses complex time-varying properties; it only limits the discussion to observation-driven models (to the exclusion of parameter-driven models), in which time-varying parameters arise as nonlinear functions of the data. An example would be the threshold models considered by [36,37], in which parameter values are allowed to differ across regimes in the data. The choice to restrict the discussion is made because it is intuitively easier to conceive of causal effects in an observation-driven context where observations represent verifiable values describing different states of real-world phenomena. At the same time, it has been shown that parametric observation-driven models can produce time-varying parameters of a wide class of nonlinear models [38] and that the forecasting power of such models may be on-par with parameter-driven models, even if the latter are correctly specified [39]. Moreover, Refs. [20,40,41] show how observation-driven models may be used to not only investigate how observations impact future observations, but also future parameter values, which may empirically be interesting if those parameters carry an economic interpretation. Finally, many popular machine learning algorithms, such as neural networks, can be reduced to equations that show how parameter values change according to levels in the data [42].

While the dynamics in Equation (1) may be nonlinear, the notation is too restrictive to nest long-memory processes. In particular, the state at time *t* is only a function of the previous state at time t−1, or t−p if the model would be generalized to *p*-order lags, but not of the full history. Vanishing dependence, implied under contraction conditions [43], is often key to verifying irreducibility and continuity [44] and proving the ergodicity of time series [45]. Proving the ergodicity of a model is needed to obtain an estimation theory under an assumption of correct specification [20,24]. Later, multivariate models will be considered, in which case long-memory properties may arise, for example, when time-varying parameters in one of the functions are a function of past data as well as of past values of those time-varying parameters.

If interrelated stochastic sequences are at the center of inference, additional building blocks are required to describe the processes. This increases the potential complexity of $P^{x}$ and $P^{y}$, but it also allows to distinguish between causality, non-causality, and feedback. Consider the stochastic system:

(2) $$\begin{array}{c} x:=\{x_{t}=f^{xx}\left(x_{t-1}\right)+f^{xy}\left(y_{t-1}\right),t\in Z\}\\ y:=\{y_{t}=f^{yx}\left(x_{t-1}\right)+f^{yy}\left(y_{t-1}\right),t\in Z\} \end{array}.$$

In this multivariate context, $f^{xy}$ and $f^{yx}$ will be referred to as the direct causal maps, while $f^{xx}$ and $f^{yy}$ control the memory properties within each channel.

When x and y are analyzed individually, the properties of $f^{xx}$ and $f^{yy}$ are of key interest. They carry information on the future positions of $x_{t+1}$ and $y_{t+1}$, and provide predictability without considering outside influence directly. However, correct causal inference around the interdependencies of x and y may be preferred over developing predictive capabilities that can result from many configurations within the parameter space that are associated with untrue probability measures. The properties of $f^{xy}$ and $f^{yx}$ determine the direction in which effects move. Verifying their properties is central to causality studies. The functions $f^{xx}$ and $f^{yy}$, on the other hand, play a central role in the system’s responses to external impulses by shaping memory of the causal initial impact of a sequence of interventions, even after that sequence turns inactive.

The functions that control memory properties within channels in some sense determine how the past reverberates into the future, and specifying correct empirical equivalents to $f^{xx}$ and $f^{yy}$ is as crucial to the inference about the causal interdependencies as is specifying mechanisms for the action of interest (it would be more general to write Equation (2) with $x:=\{x_{t}=f^{xx}\left(x_{t-1};w_{t-1}\right)+f^{xy}\left(y_{t-1};w_{t-1}\right),t\in Z\}$ and $y:=\{y_{t}=f^{yx}\left(x_{t-1};w_{t-1}\right)+f^{yy}\left(y_{t-1};w_{t-1}\right),t\in Z\}$ and with $w_{t}=\left(x_{t},y_{t}\right)$. In this case, for instance, the dependence of $x_{t}$ on its own past, $x_{t-1}$, is allowed to vary based on the levels in past data. However, under this notation, one could at any point in time, decompose the change in one variable into effects attributed to memory and outside influence separately, which the simplified notation in Equation (2) is intended to focus on). In fact, as Ref. [46] point out, systems may be dominated by memory and the influence of the causal components may be small on the overall process in which case predictive power can be obtained without specifying any causal maps and focusing solely on memory. Inversely, this also suggests that one must obtain a model for the memory process to isolate the causal impacts themselves, suggesting that long-memory applications in which causal inference is of interest must develop a high degree of predictive power, even if prediction is not needed for policy purposes. This can be made more clear by considering the following:

(3) $$\begin{array}{c} x^{0}:=\left\{x_{t}^{0}=f^{xy}\left(y_{t-1}\right),t\in Z\right\}\\ y^{0}:=\left\{y_{t}^{0}=f^{yx}\left(x_{t-1}\right),t\in Z\right\} \end{array},$$

with $x^{0}$ and $y^{0}$ defined as $x_{t}^{0}=x_{t}-f^{xx}\left(x_{t-1}\right)$ and $y_{t}^{0}=y_{t}-f^{yy}\left(y_{t-1}\right)$. Given the realized sequences y(ω) and x(ω) generated by Equation (2), the sequential system of Equation (3) moves forward in time as the one-step-ahead directly caused parts of y and x that are filtered from the reverberating effects of $f^{xx}$ and $f^{yy}$. More specifically, while y partially consists of memory, there is a part, $y^{0}$, that, at any point, is directly mapped from the previous state of x, while, at the same time, x consists partially of memory and a part $x^{0}$ directly generated from the last position of y. In this view, directional causality can be stated in terms of whether (3) produces any values, i.e., diagnosing if there is any statistically significant signal from initial causal impulses left after all memory properties have been stripped from the data. Importantly, the system reveals that by the definitions of $x_{t}^{0}$ and $y_{t}^{0}$, obtaining appropriate estimates for $f^{xy}$ and $f^{yx}$ involves $f^{xx}$ and $f^{yy}$ being modeled correctly as $x_{t}^{0}$ and $y_{t}^{0}$ are not observed and only result as functions from the observable processes y and x. Moreover, if y(ω) and x(ω) are triggered by an event, then it is possible, by process of infinite backward substitution, to write Equation (3) as an infinite chain initialized in the infinite past. Plugging in the equalities $x_{t}=x_{t}^{0}+f^{xx}\left(x_{t-1}\right)$ and $y_{t}=y_{t}^{0}+f^{yy}\left(y_{t-1}\right)$ and defining the random functions $f_{y}^{0}\left(y_{t}^{0},y_{t-1}\right)=f^{xy}\left(y_{t}^{0}+f^{yy}\left(y_{t-1}\right)\right)$ and $f_{x}^{0}\left(x_{t}^{0},x_{t-1}\right)=f^{yx}\left(x_{t}^{0}+f^{xx}\left(x_{t-1}\right)\right)$, one can write

(4) $$\begin{array}{c} x^{0}:=\left\{x_{t}^{0}=f_{y}^{0}\left(y_{t-1}^{0},y_{t-2}\right),t\in Z\right\}\\ y^{0}:=\left\{y_{t}^{0}=f_{x}^{0}\left(x_{t-1}^{0},x_{t-2}\right),t\in Z\right\} \end{array}.$$

Repeating infinitely, and extending infinitely in the direction T→∞,

(5) $$\begin{array}{c} x^{0}:=\left\{x_{\infty }^{0}={\left(f_{y}^{0}\right)}^{\infty }\left(y_{1}^{0},y_{1}\right),t\in Z\right\}\\ y^{0}:=\left\{y_{\infty }^{0}={\left(f_{x}^{0}\right)}^{\infty }\left(x_{1}^{0},x_{1}\right),t\in Z\right\} \end{array}.$$

${\left(f_{y}^{0}\right)}^{\infty }$ and ${\left(f_{x}^{0}\right)}^{\infty }$ are the maps that generate $y^{0}$ and $x^{0}$ infinitely after y and x have been generated into infinity. Subscript ${}_{1}$ has been used, here, to mark the initialization points. This shows that $x^{0}$ can be written as a sequence of iterating functional operations that are all defined on y, and $y^{0}$ defined on x in a similar way (Equation (5) reveals that the sequences that constitute the directly caused parts of x and y are ultimately dependent on the values at which the observable process has been initialized. That is, the entire causal pathway depends on the initial impact. In practice, one cannot observe all impacts—including those that occurred in the infinite past—and assurance is required that the initialization effect of the causal pathway must, asymptotically, be irrelevant). For ease of notation, let us write

(6) $$\begin{array}{c} x^{0}:=\left\{x_{t}^{0}=f_{y}^{0}\left(y_{-\infty :t}\right),t\in Z\right\}\\ y^{0}:=\left\{y_{t}^{0}=f_{x}^{0}\left(x_{-\infty :t}\right),t\in Z\right\} \end{array}.$$

where bold-faced $f^{0}$ is used to refer to the entire sequence of functional operations $f^{0}$ up to *t*, starting in the infinite past t=−∞. This highlights that generating the unobserved quantities $x^{0}$ and $y^{0}$ from the observed quantities x and y by back substitution eventually involves the unobserved quantities $x_{1}$ and $y_{1}$. This means that some feasible form of approximation is needed, since time series data in practice area almost never recorded since the beginning of the process.

Note first that $f_{y}^{0}:Y\to X\subseteq R$ is a B(Y)/B(X)-measurable mapping, and $f_{x}^{0}:X\to Y\subseteq R$ is a B(X)/B(Y)-measurable mapping. The sequence $x^{0}$ thus lives on $\left(X_{\infty },B\left(X_{\infty }\right),P_{0}^{x}\right)$, where $P_{0}^{x}$ is induced according to $P_{0}^{x}\left(B_{x}\right)=P^{y}\;○\;{\left(f_{y}^{0}\right)}^{-1}\left(B_{x}\right)\;\forall \;B_{x}\in B\left(X_{\infty }\right)$, and $y^{0}$ lives on $\left(Y_{\infty },B\left(Y_{\infty }\right),P_{0}^{y}\right)$, where $P_{0}^{y}$ is induced according to $P_{0}^{y}\left(B_{y}\right)=P^{x}\;○\;{\left(f_{x}^{0}\right)}^{-1}\left(B_{y}\right)\;\forall \;B_{y}\in B\left(Y_{\infty }\right)$, see [47] p. 118 and [48] p. 115. The notation shows that the probability measures underlying the stochastic causal sequences result from the functional behavior of the entire system. In particular, the causal sequences can be written as recursive direct effects from another variable that itself consists of memory and causal effects, and the probability measures underlying the causal sequences are thus induced by the functional relationships that describe all dynamical dependencies. This is important to the extent that many causal studies focus on one single marginal dependency, while, from the measure-theoretic perspective developed here, the wider system within any one single process operates, is of importance to the analysis. This suggests that researchers must pay attention to referencing the workings of a broader system when designing their models for inference, something [33] has also argued. Moreover, it has been argued (see [49] for discussion) that probabilistic definitions of causality are not strictly causal in the sense that they do not provide insight in the origin of the probability law that regulates the process of interest, and that a (correct) time-series model only describes (correctly) the probabilistic behavior as the outcome of that unknown causal origin. The notation, here, shows, however, explicitly the relation between the functional behavior of a system and its induced probability measure that assigns probability to all possible outcomes. This suggests that such critiquing views, rather, relate to disagreements around the level of detail in the structure of a model, which in turn would be guided by the research question of interest and the availability of detailed data. Particularly, dynamical systems in economics are often modeled using aggregate macro-economic data that do not have the same granularity as micro-economic data containing information about the behaviors of individual economic agents.

In many cases, a researcher is not able to observe all the relevant variables. When a third, possibly unobserved external variable, z, with effect $f^{z}\left(z\right)$, is considered, the researcher is confronted with the situation that

(7) $$\begin{array}{c} x:=\{x_{t}=f^{xx}\left(x_{t-1}\right)+f^{xy}\left(y_{t-1}\right)+f^{xz}\left(z_{t-1}\right),t\in Z\}\\ y:=\{y_{t}=f^{yx}\left(x_{t-1}\right)+f^{yy}\left(y_{t-1}\right)+f^{yz}\left(z_{t-1}\right),t\in Z\} \end{array}.$$

If z is unobserved, it can still be approximated as a difference combination of x and y. To obtain an approximated sequence of the *true* z sequence to condition empirical counterparts for $f^{xz}$ and $f^{yz}$ on, one can work with:

(8) $$\begin{array}{c} z:=\{z_{t}=f^{z|xy}\left(x_{t+1}-\left(f^{xx}\left(x_{t}\right)+f^{xy}\left(y_{t}\right)\right)\right),t\in Z\}\\ z:=\left\{z_{t}=f^{z|yx}\left(y_{t+1}-\left(f^{yx}\left(x_{t}\right)+f^{yy}\left(y_{t}\right)\right)\right),t\in Z\right\} \end{array}.$$

Equation (8) suggests to write Equation (7) in terms of y and x only by defining z as a difference combination of x and y (Apart from stability conditions imposed on the endogenous process, one requires also that the exogenous impacts enter the system in some suitable manner, which, for example, requires that $f^{xz}$ and $f^{yz}$ are appropriately bounded. Following the same arguments that resulted in Equation (5), the initialization of the exogenous impacts $z_{1}$ should similarly not carry information influential in the empirical estimates of $f^{xy}$ and $f^{yx}$, conditional on partial information). This allows us to define the spaces and measures in terms of x and y when the multivariate process includes further variables, in this case, z. If the process is invertible, one can write, by aggregating the functions:

(9) $$\begin{array}{c} x:=\{x_{t}=f^{xx}\left(x_{t-1}\right)+f^{xy}\left(y_{t-1}\right)+f^{xz}\left(x_{t},x_{t-1},y_{t-1}\right),t\in Z\}\\ y:=\{y_{t}=f^{yx}\left(x_{t-1}\right)+f^{yy}\left(y_{t-1}\right)+f^{yz}\left(y_{t},x_{t-1},y_{t-1}\right),t\in Z\} \end{array}.$$

(10) $$\begin{array}{c} x:=\{x_{t}=f^{x}\left(x_{t-1},y_{t-1}\right),t\in Z\}\\ y:=\{y_{t}=f^{y}\left(x_{t-1},y_{t-1}\right),t\in Z\} \end{array}.$$

(11) $$\begin{array}{c} x:=\{x_{t}=f^{x}\left(w_{t-1}\right),t\in Z\}\\ y:=\{y_{t}=f^{y}\left(w_{t-1}\right),t\in Z\} \end{array}.$$

For every t∈Z, the map $f^{x}\;○\;\left(y_{t-1},x_{t-1}\right):\Omega \to X$ is F/B(X)-measurable and x(ω) lives on the space $\left(X_{\infty },B\left(X_{\infty }\right),P^{x}\right)$ where the probability measure $P^{x}$ is induced by $f^{x}$ on $B(X_{\infty })$ according to the point-wise application of $P^{w}$ and the inverse of $f^{x}$. ($P^{x}\left(B_{x}\right)=P^{w}\;○\;(f^{x}{)}^{-1}\left(B_{x}\right)\;\forall \;\left(B_{x}\right)\in B\left(X_{\infty }\right)$). Similar arguments follow for $P^{y}$. This tells us that, in the general case of multivariate dependencies and in the presence of possibly unobserved variables, the probability measures underlying the individual sequences are possibly a result of those of the other sequences. This means the space of empirical candidates for the probability measure $P^{w}$ that underlies the joint process $w:=\{w_{t}=\left(y_{t},x_{t}\right),t\in Z\}$ operates on $\left(W_{\infty },B\left(W_{\infty }\right),P^{w}\right)$. (The sequence realizes under the events ω∈Ω, $w_{t}\left(\omega \right)\in W$, where W:=Y×X and $w\left(\omega \right)\in W_{\infty }$, with $W_{\infty }:=Y_{\infty }\times X_{\infty }\subseteq R_{\infty }^{n_{x}+n_{y}}:=\times_{t=-\infty }^{t=\infty }R^{n_{x}+n_{y}}$, and the probability measure of the joint process $P^{w}$ is thus defined on the product σ-algebra $B\left(W_{\infty }\right)=B\left(X_{\infty }\times Y_{\infty }\right)=B\left(X_{\infty }\right)⊗B\left(Y_{\infty }\right):=W_{\infty }\cap B\left(R_{\infty }^{n_{x}+n_{y}}\right)$ (see, [47] p. 119)).

Regardless, the measure $P^{w}$ is induced by functional relations of Equation (2), which, as was shown, can be decomposed into memory and causal subsystems. One can thus state causality conditions, based on the measures that describe the directly caused effects represented by Equation (6). In particular, one can keep the focus on $P_{0}^{x}$ and $P_{0}^{y}$, bearing in mind that they are lower-level constituents of $P^{w}$ on which, in turn, the complete estimation objective will be defined.

**Definition** **1** (Non-causality)**.** *The stochastic sequences x(ω) and y(ω) are not causally related if $P_{0}^{x}$ and $P_{0}^{y}$ are null measures, such that $x^{0}\left(\omega \right)\in \emptyset \;\forall \;\left(\omega ,t\right)\in \Omega \times Z$ and $y^{0}\left(\omega \right)\in \emptyset \;\forall \;\left(\omega ,t\right)\in \Omega \times Z$.*

**Definition** **2** (Uni-directional Causality)**.** *Causality runs uni-directionally from the stochastic sequence x(ω) to another stochastic sequence y(ω) (visa versa), if $P_{0}^{x}$ is a null measure, and $P_{0}^{y}$ is a non-null measure, such that $x^{0}\left(\omega \right)\in \emptyset \;\forall \;\left(\omega ,t\right)\in \Omega \times Z$ and $y^{0}\left(\omega \right)\in Y\;\forall \;\left(\omega ,t\right)\in \Omega \times Z$ (visa versa).*

**Definition** **3** (Bi-directional Causality)**.** *The stochastic sequence x(ω) is causal with respect to y(ω) and y(ω) is causal with respect to x(ω), if $P_{0}^{x}$ and $P_{0}^{y}$ are both non-null measures, such that $x^{0}\left(\omega \right)\in X\;\forall \;\left(\omega ,t\right)\in \Omega \times Z$ and $y^{0}\left(\omega \right)\in Y\;\forall \;\left(\omega ,t\right)\in \Omega \times Z$.*

Respectively, conditioning on impacts in x, these probabilistic causality definitions can thus be understood broadly as:

1. Whenever an intervention in x occurs, there is no chance that $y^{0}$ reacts as a result of that.
2. Whenever an intervention in x occurs, there is positive chance that $y^{0}$ reacts as a result of that.
3. Whenever an intervention in x occurs, there is positive chance that $y^{0}$ reacts as a result of that. Subsequently there is positive chance that x reacts to this initial reaction, a probabilistic process that repeats recursively.

**Remark** **1.** *With null-measures, it is meant that the stochastic sequence describing the directly caused effects from one variable to the other takes values in the empty set with probability 1. This is because the functions that induce the probability measure cancel out, hence, they can be removed from the equations resulting in a probability measure that is not induced by any remaining rule or relationship. In practice, one can test whether $P^{x}\left|f^{xx}\equiv P^{x}\right|f^{xx}f^{xy}$ or $P^{x}\left|f^{xx}\equiv P^{x}\right|f^{xx}f^{xy}$, where $P^{x}|f^{xx}$ here denotes the probability measure induced by the functional relationships in Equation (1) and $P^{x}|f^{xx}f^{xy}$ denotes the probability measure induced by the functional relationships in Equation (2), to test whether $P_{0}^{x}$ exists. A practical test is a Kolmogorov–Smirnov-type test.*

## 3. Limit Divergence on the Space of Modeled Probability Measures

The definitions of causality, in terms of the lower-level components of $P^{w}$, suggest that correct causal statements can be obtained empirically by extracting relevant counterparts to $P_{0}^{x}$ and $P_{0}^{y}$ from a relevant counterpart to $P^{w}$, and investigating the stochastic sequences produced by these modeled measures. For such an approach to be of relevance in an empirical context, one must ensure that the concepts introduced adequately transfer over from the *true* measure $P^{w}$ to a modeled measure $P^{\hat{w}}$. The focus is therefore shifted towards detailing how $P^{\hat{w}}$ can be approximated as a minimally divergent measure relative to $P^{w}$, and draw on approximation theory to construct equivalence around the *true* measure under an axiom of correct specification.

For some event ω∈Ω, a realized *T*-period sequence $w_{T}\left(\omega \right):=\left(y_{T}\left(\omega \right),x_{T}\left(\omega \right)\right)$ consisting of sequences ${\left\{y_{t}\left(\omega \right)\right\}}_{t=1}^{t=T}$ and ${\left\{x_{t}\left(\omega \right)\right\}}_{t=1}^{t=T}$ can be observed. The *true* function $f^{w}$, consists of our main functions of interest $f^{x}$ and $f^{y}$ that in turn are composed of $f^{xy}$ and $f^{yx}$ that are of particular interest to the researcher focused on causality, but possibly also functions $f^{xx}$ and $f^{yy}$ that shape the responses of an initial causal effect. The exact properties are generally unknown to the observer, but one can design a parameterization mapping that learns the behavior of $f^{x}$ and $f^{y}$ when exposed to sufficient data. To learn from the data an approximation of $f^{x}$ and $f^{y}$, one can postulate a model

(12) $$\hat{w}:=\left\{{\hat{w}}_{t}=f\left(w_{t-1};\theta \right),\theta \in \Theta ,t\in Z\right\},$$

with *f*:W×Θ→W as our postulated model function and $\hat{w}$ as the modeled data. In the context of parametric inference, the parameter space Θ is of finite dimensionality, but also in the nonparametric case, the vector θ∈Θ indexes parametric models nested by the nonparametric model, each inducing its own probability measure, and Θ indexes families of parametric models, each inducing a space of parametric functions generated under Θ. In this discussion a compact set of potential hypotheses is considered, limiting the inference to parametric models. The arguments can be extended to the nonparametric case, by focusing on a compact subset $\Theta_{s}\subset \Theta$ of solutions (For example, by letting $\Theta_{s}$ grow as T→∞, hence focusing on the case $\Theta_{s1}\subset \Theta_{s2}\ldots \subset \Theta_{s\infty }\subseteq \Theta$, see for example [50]). For example, by using priors or penalties that discard $\Theta \backslash \Theta_{s}$ such that any solution of the criterion necessarily falls within a compact subset space, see [20] p. 210 and [24]. Let *f* be B(W)-measurable ∀θ∈Θ so that $f(w_{t};\theta ):\Omega \to W$ is F/B(W)-measurable ∀θ∈Θ and t∈Z. $F_{\Theta }:=\left\{f\left(\cdot ;\theta \right),\theta \in \Theta \right\}$ is our space of parametric functions defined on W generated under Θ under the injective $f_{W}:\Theta \to F_{\Theta }\left(W\right)$ where $f_{W}\left(\theta \right):=f\left(\cdot ;\theta \right)\in F_{\Theta }\left(W\right)\;\forall \;\theta \in \Theta$. Under any *true* probability measure $P^{w}$, every potential parameter vector included in the parameter space θ∈Θ induces a probability measure $P_{\theta }^{\hat{w}}$ indexed by θ on $B(W_{\infty })$, according to $P_{\theta }^{\hat{w}}\left(B_{w}\right)=P^{w}\;○\;f^{-1}\left(B_{w},\theta \right)\;\forall \;\left(B_{w},\theta \right)\in B\left(W_{\infty }\times \Theta \right)$. Thus, for every potential parameter vector included in the parameter space θ∈Θ, there is a triplet $\left(W_{\infty },B\left(W_{\infty }\right),P_{\theta }^{\hat{w}}\right)$ that describes the probability space of modeled data under θ. The triplet $\left(W_{\infty },B\left(W_{\infty }\right),P_{\theta }^{\hat{w}}\right)$ is, thus, itself an element of the measure spaces indexed by θ across all Θ. Given the *true* probability measure $P^{w}$ on B(W), this process is summarized by a functional $P:F_{\Theta }\left(W\right)\to P_{\Theta }^{\hat{w}}$, that maps elements from the space of parametric functions generated by the entire parameter space $F_{\Theta }\left(W\right)$, onto the space $P_{\Theta }^{\hat{w}}$ of probability measures defined on the sets of $B(W_{\infty })$ generated by Θ through f(·;θ).

Now, $f^{w}$ is generally not only unknown, but for a finite Θ there is no guarantee that $\exists \theta_{0}\in \Theta :P\;○\;f_{W}\left(\theta_{0}\right)=P^{w}$, implying that, in many empirical applications, one is concerned with the situation where $P^{w}\notin P_{\Theta }^{\hat{w}}$. However, if $\exists P^{w}\in P_{\Theta }^{\hat{w}}$, one can learn all about $P^{w}$ by uncovering the properties of *f*, given that a sufficient amount of observations is available. (As discussed in the literature on miss-specification, even when the axiom of correct specification is abandoned, *f* may converge to a function that produces the optimal conditional density, which may reveal properties of $f^{w}$). Let

(13) $${\hat{\theta }}_{T}:=\arg\min_{\theta \in \Theta }Q_{T}\left(w_{T};\theta \right),$$

${\hat{\theta }}_{T}:\Omega \to \Theta$, be the extremum estimate for $\theta_{0}$ as judged by the criterion $Q_{T}:W_{T}\times \Theta \to R$. Trivially, $W_{T}:=Y_{T}\times X_{T}$ and $w_{T}\left(\omega \right)\in W_{T}$. To see that under correct specification it is possible to approximate the *true* function $f^{w}$ in terms of equivalence (in the sense of function equivalence [51] p. 288), one can write the criterion function also as a function of the *true* function and the postulated model $Q_{T}\left(f^{w}\left(w_{T}\right),f\left(w_{T};\theta \right)\right)$ in which it is made use of the fact that $f^{w}\left(w_{T}\right):={\left\{f^{w}\left(w_{t}\right)\right\}}_{t=1}^{T}:=w_{T}$ and $f\left(w_{T};\theta \right):={\left\{f\left(w_{t};\theta \right)\right\}}_{t=1}^{T}:={\hat{w}}_{T}$.

The discussion further evolves toward showing that the element in $P_{\Theta }^{\hat{w}}$ that is closest to $P^{w}$ minimizes a divergence metric that results from a transformation of the limit criterion that measures the divergence between the *true* density and the density implied by the model. Note that $P_{\Theta }^{\hat{w}}$ is induced by the proposed candidates for $P^{w}$; studies on causality thus rely on flexible model design as the researcher determines which hypotheses are considered in a study by exerting control over Θ. Naturally, if $\Theta_{1}\subset \Theta_{2}$, then $\Theta_{2}$ produces a larger $P_{\Theta_{2}}^{\hat{w}}⊃P_{\Theta_{1}}^{\hat{w}}$. This suggests that minimizing this divergence metric over a large as possible $P_{\Theta }^{\hat{w}}$ results in selecting $P^{\hat{w}}$ at a point in $P_{\Theta }^{\hat{w}}$ that attains equivalence to $P^{w}$ only when Θ is large enough to produce a correctly specified hypothesis set. Note that the definition of $F_{\Theta }:=\left\{f\left(\cdot ;\theta \right),\theta \in \Theta \right\}$, as our space of parametric functions generated under Θ, under the injective $f_{W}:\Theta \to F_{\Theta }\left(W\right)$ and the functional $P:F_{\Theta }\left(W\right)\to P_{\Theta }^{\hat{w}}$ that induces the space of probability measures, is defined on the sample space W. This highlights that the correct specification argument, $P^{w}\in P_{\Theta }^{\hat{w}}$, not only stresses flexible parameterization in the sense that parameterized dependencies can take on many values, but also in the sense of using correct data (Indeed, the potential parameters that would interact with data that is not used are essentially treated as zero, so the focus on using correct data is implicitly already contained in the standard statements of correct specification that focus directly on the dimensions of Θ. The distinction is nevertheless useful because nonparametric models are often popularized as methods to reduce miss-specification bias as Θ becomes infinite dimensional, but this does not imply that $P^{w}\in P_{\Theta }^{\hat{w}}$ if important data is missing). When little is known about *f*, one is thus not only concerned with flexibility in terms of the type of parametric functions generated under Θ, but also the variables on which the modeled measures are defined. When these concerns are appropriately addressed, testing for causality is deciding based on the approximation $P^{\hat{w}}$ whether the best approximation of the *true* model suggests (1) that x and y live in isolation, (2) unidirectional causality, or (3) that $P^{w}$ produces feedback.

To turn this problem into a selection problem that can be solved by divergence minimization w.r.t. the *true* measure, first introduce the limit criterion by taking T→∞ and working with the modeled data as the minimizer of the criterion. Specifically, let the limit criterion be $Q_{\infty }\left(\theta \right):=Q_{T}\left(f^{w}\left(w_{T}\right),f\left(w_{T};\arg\min_{\theta \in \Theta }Q_{T}\left(w_{T};\theta \right)\right)\right)$ evaluated at T→∞ with $Q_{\infty }:\Theta \to R$ and $Q_{\infty }\left(\theta \right)=Q_{\infty }^{P}\left(P^{w};P_{\theta }^{\hat{w}}\right)\;\forall \;\theta \in \Theta$ with the criterion $Q_{\infty }\left(\theta \right)=Q_{\infty }^{P}$ as a measure of divergence $d_{P}$ on the *true* probability measure and the modeled measure. More specifically, $d_{P}\equiv Q_{\infty }^{P}:P_{\Theta }^{\hat{w}}\times P_{\Theta }^{\hat{w}}\to R_{\geq 0}$. By definition of $Q_{\infty }^{P}$ as a divergence on the space that contains $P^{w}$ and $P_{\theta }^{\hat{w}}\;\forall \;\theta \in \Theta$, the element $\theta_{0}$ is thus the minimizer of that divergence.

Moreover, argmin in the parameter sense, argmin in the function sense (in terms of a divergence metric on the *true* function), and argmin in the measure sense (in terms of a divergence metric on the *true* probability measure), are equivalent limits under the same consistency result. To see this, it is convenient to focus once more on the target and write $\theta_{0}=\arg\min_{\theta \in \Theta }Q_{\infty }^{P}\equiv \arg\min_{\theta \in \Theta }Q_{\infty }^{F}\left(f^{w},f_{W}\left(\theta \right)\right)$, with $Q_{\infty }^{F}:F\left(W\right)\times F\left(W\right)\to R_{\geq 0}$, to make clear that the criterion establishes a divergence $d_{F}$ on F(W)×F(W), which is, in turn, induced by $d_{P}$ through P according to $d_{F}\left(f^{1},f^{2}\right)=d_{P}\left(P\left(f^{1}\right),P\left(f^{2}\right)\right)\;\forall \;\left(f^{1},f^{2}\right)\in F\left(W\right)\times F\left(W\right)$. This ensures that our statement on the probability measure is relevant under standard consistency results that are focused on the convergence of an estimated parameter vector toward $\theta_{0}$, while, equivalently, the impulse response functions (IRFs) converge to the *true* IRFs at $\theta_{0}$. This implies that deciding between Definitions 1–3 can be read from the responses produced by the IRF that minimizes divergence w.r.t. the *true* IRF.

Not necessary, but convenient for a proof that holds easily in practical situations, is to assume the existence of a strictly increasing function $r:R\to R_{\geq 0}$ that ensures the existence of a transformation of the limit criterion into a metric, $d_{P}^{*}\equiv r\;○\;d_{P}$, with *r* being a continuously and strictly increasing function. For convenience, all assumptions are summarized in Assumption 1.

**Assumption** **1.** For a limit criterion $Q_{\infty }:\Theta \to R$ of the form $Q_{\infty }\left(\theta \right)\equiv Q_{\infty }^{P}\left(P^{w},P_{\theta }^{\hat{w}}\right)\;\forall \;\theta \in \Theta$, $d_{P}\equiv Q_{\infty }^{P}:P^{w}\times P^{w}\to R_{\geq 0}$ is a divergence. Assume there exists a continuous strictly increasing function $r:R\to R_{\geq 0}$ such that $d_{P}^{*}\equiv r\;○\;d_{P}$ is a metric. The functional $f_{W}:\Theta \to F_{\Theta }\left(W\right)$ is injective and $\theta_{0}\in \Theta$.

**Proposition** **1.** *Assume 1, then the following are equivalent limits:*

1. *$\theta_{0}$,*
2. *$\arg\min_{\theta \in \Theta }Q_{\infty }\left(\theta \right)$,*
3. $\arg\min_{\theta \in \Theta }d_{F}^{*}\left(f^{w},f^{\hat{w}}\left(\cdot ,\theta \right)\right)$,
4. *$\arg\min_{\theta \in \Theta }Q_{\infty }^{P}\left(P^{w},P_{\theta }^{\hat{w}}\right)$,*
5. $\arg\min_{\theta \in \Theta }d_{P}^{*}\left(P^{w},P_{\theta }^{\hat{w}}\right)$.

**Remark** **2.** *Dropping the axiom of correct specification implies ${\hat{\theta }}_{\infty }\neq \theta_{0}$, hence, the equivalences of 3–5 are now w.r.t. item 2.*

The equivalences in Proposition 1 not only ensure that for a correctly specified model $\exists \theta_{0}\in \Theta$, the element $\theta_{0}$ results in functional equivalence between the model and the *true* model (item 3), but also in zero divergence between the probability measures $P^{w}$ and $P_{\theta }^{\hat{w}}$ (item 4). Moreover, it follows that at $\theta_{0}$, the empirically estimated probability measure $P^{\hat{w}}$ is equivalent to $P^{w}$ in the sense that there is zero distance between the two (item 5).

**Remark** **3.** *Proposition 1 is applicable to a large class of extremum estimators, even those not initially conceived as minimizers of distance. In particular it is often possible to find a divergence on the space of probability measures. For example, method of moments estimators are naturally defined in terms of features of the underlying probability measures. In Section 4 and example is given, using Kullback–Leibler divergence, for which penalized likelihood is an estimator. In this case squared Hellinger distance can be shown to be a lower bound.*

Corollary 1 now delivers that our definitions, set on the *true* measures, transfer to modeled probability measures in the limit for correctly specified cases. It is well-known that standard consistency proofs apply also to approximate extremum estimators, therefore, assuming additionally that $\sup_{\theta \in \Theta }\left|Q_{T}\left(w_{T};\theta \right)-Q_{\infty }\left(\theta \right)\right|\to 0$*a.s.*, is sufficient for a consistency result together with the uniqueness of $\theta_{0}$ within the compact hypothesis space Θ (Note that, under the axiom of correct-specification, consistency results require suitable forms of stability defined on the process rather than the data. While we have loosely remarked on the fact that the non-parametric case of an infinite dimensional Θ is easily allowed, stability of highly nonlinear multivariate time series is a difficult separate topic. Regardless, Refs. [44,45] provide Ergodicity results for a large class of nonlinear time series that include non-parametric ones. The conditions require the nonlinearities to be sufficiently smooth. Specific stability results have also been established for certain neural network models, for example by [52]). This implies that our causality conditions on the *true* measures do not only transfer to the approximate in the limit, but also for large *T* under standard regularity conditions. Essentially, this is the setting considered by Ref. [11]. Summarized:

**Corollary** **1.** *Given a* true probability measure $P^{w}$, and an equivalent modeled probability measure $P^{\hat{w}}$ in the sense that $d_{P^{\hat{w}}}^{*}=r\;○\;d_{P}\left(P^{w},P_{\theta }^{\hat{w}}\right)∼0$, there are four possibilities for causality:

*1*. *There is no causation if $P_{0}^{\hat{x}}$ and $P_{0}^{\hat{y}}$ adhere to Definition 1.*
*2*. *x causes y if the probability measure $P_{0}^{\hat{y}}$ adheres to Definition 2.*
*3*. *y causes x if the probability measure $P_{0}^{\hat{x}}$ adheres to Definition 2.*
*4*. *There is bi-directional causality if $P_{0}^{\hat{x}}$ and $P_{0}^{\hat{y}}$ adhere to Definition 3.*

Finally, in the case of a miss-specified model, Proposition 2 implies that the divergence between the optimal probability measure as judged by the criterion and the *true* probability measure attains a minimum at a strictly positive value $d_{P^{w}}^{*}>0$. In this case, the quantity $d_{P^{\hat{w}}}^{*}$ determines how “close” the empirical claim is to the *true* hypothesis about causality. While it is difficult to make claims about this quantity, it is evident that minimizing $d_{P^{\hat{w}}}^{*}$ may involve widening $P_{\Theta }^{\hat{w}}$ in the direction of $P^{w}$ by increasing the dimensionality of Θ and allow flexibility while investigating a wide range of data. Disregarding the value of $d_{P^{\hat{w}}}^{*}$, the following holds.

**Proposition** **2.** *If $\theta_{0}\notin \Theta$, then $P^{w}\notin P_{\Theta }^{\hat{w}}$. However, ${\hat{\theta }}_{\infty }$ is still the pseudo-*true *parameter that minimizes $r\;○\;d_{P}\left(P^{w},P_{\theta }^{\hat{w}}\right)$ over Θ. Therefore, $P^{\hat{w}}$ is the probability measure minimally divergent from $P^{w}$ within $P_{\Theta }^{\hat{w}}$. As such, it follows that, from all the potential probability measures in $P_{\Theta }^{\hat{w}}$, the measure closest to $P^{w}$ is supportive of one out of 1−4 in corollary 1 based on the properties of $P_{0}^{\hat{x}}$ and $P_{0}^{\hat{y}}$ as the best approximations. $P^{\hat{w}}$ provides the best approximation of the* true *causal measure across all the hypotheses considered.*

This leads to the following collection of results.

**Corollary** **2.** *Given a* true *probability measure $P^{w}$, and a non-equivalent, but pseudo-*true modeled probability measure, $P^{\hat{w}}$, in the sense that $d_{P^{w}}^{*}=r\;○\;d_{P}\left(P^{w},P_{\theta }^{\hat{w}}\right)$ has attained a non-zero minimum, there are four possible optimal hypotheses about causality, as judged by the criterion:

*1*. *There is no causation if $P_{0}^{\hat{x}}$ and $P_{0}^{\hat{y}}$ adhere to Definition 1.*
*2*. *x causes y if the probability measure $P_{0}^{\hat{y}}$ adheres to Definition 2.*
*3*. *y causes x if the probability measure $P_{0}^{\hat{x}}$ adheres to Definition 2.*
*4*. *There is bi-directional causality if $P_{0}^{\hat{x}}$ and $P_{0}^{\hat{y}}$ adhere to Definition 3.*

Respectively, conditioning on interventions in x, the results can be understood as:

1. Whenever an intervention in x occurs, our best hypothesis is that there is no chance that y reacts as a result of that.
2. Whenever an intervention in x occurs, our best hypothesis is that there is positive chance that y reacts as a result of that.
3. Whenever an intervention in x occurs, our best hypothesis is that there is positive chance that y reacts as a result of that, and these interactions continue to repeat with positive probability.

## 4. Limit Squared Hellinger Distance

Both Corollaries 1 and 2 assume that an appropriate transformation of the limit criterion exists that provides us with a metric or norm. This assumption allows us to make use of the classical theorems on existence and uniqueness of best approximations that have been naturally obtained for metric, normed, and inner product spaces [53]. While this retains the simplicity of the argument, it also shows that a direct interpretation of Corollaries 1 and 2 can be obtained within the framework of maximum likelihood. Let us first define the criterion function as the maximum likelihood estimator:

(14) $$\arg\min_{\theta \in \Theta }Q_{T}\left(w_{T};\theta \right):=\arg\max_{\theta \in \Theta }\sum_{t=1}^{T}\ln p_{t}\left(w_{t}|\theta \right).$$

Note that this is conforming to $Q_{\infty }\left(\theta \right):=Q_{T}(f^{w}\left(w_{T}\right),f(w_{T};\arg\min_{\theta \in \Theta }$$Q_{T}\left(w_{T};\theta \right)))$ with T→∞ and $Q_{\infty }:\Theta \to R$. It can be shown that, under this definition with $Q_{\infty }\left(\theta \right)=Q_{\infty }^{P}\left(P^{w};P_{\theta }^{\hat{w}}\right)\;\forall \;\theta \in \Theta$, the criterion $Q_{\infty }\left(\theta \right)=Q_{\infty }^{P}$ is a measure of divergence $d_{P}$ on the *true* probability measure and the modeled measure. Specifically, we can introduce a divergence $d_{P}\equiv Q_{\infty }^{P}:P^{w}\times P^{w}\to R_{\geq 0}$ as follows. Let $p^{w}\left(w_{t}|\theta_{w}\right)$ and $p^{\hat{w}}\left(w_{t}|\theta_{\hat{w}}\right)$ be, respectively, the *true* density evaluated under the *true* parameter and a modeled density at $\hat{\theta }$, evaluated under the estimated parameter, both at time *t*, with respect to the Lebesque measure (such that they are probability density functions); then the following is a divergence from the true probability measure to the modeled probability measure (Kullback–Leibler divergence, see [54]):

(15) $$\begin{array}{c} KL\left(P^{w}\left(w|\theta_{w}\right)\left|\right|P^{\hat{w}}\left(w|\theta_{\hat{w}}\right)\right)=\\ \;\;\;\;\;\;\;\;\left\{\begin{array}{cc} \int_{-\infty }^{\infty }p^{w}\left(w|\theta_{w}\right)\ln\frac{p^{w}\left(w|\theta_{w}\right)}{p^{\hat{w}}\left(w|\theta_{\hat{w}}\right)}dw & \forall \;p^{w}\left(w|\theta_{w}\right)\ll p^{\hat{w}}\left(w|\theta_{\hat{w}}\right)\\ \infty & \mathrm{otherwise} \end{array}\right.. \end{array}$$

Naturally, $KL\left(P^{w}\left(w|\theta_{w}\right)\left|\right|P^{\hat{w}}\left(w|\theta_{\hat{w}}\right)\right)\geq 0$ with equality if and only if $p^{w}\left(w|\theta_{w}\right)$$=p^{\hat{w}}\left(w|\theta_{\hat{w}}\right)$ almost everywhere, i.e., when the probability measures are the same (this is known as Gibb’s inequality and can be verified by applying Jensen’s inequality).

Kullback–Leibler divergence is not a distance metric, as was used in Corollaries 1 and 2 to establish equivalences by partitioning into classes of zero-distance points. In particular, it is asymmetric

(16) $$KL\left(P^{w}\left(w|\theta_{w}\right)\left|\right|P^{\hat{w}}\left(w|\theta_{\hat{w}}\right)\right)\neq KL\left(P^{\hat{w}}\left(w|\theta_{\hat{w}}\right)\left|\right|P^{w}\left(w|\theta_{w}\right)\right),$$

and the triangle inequality is also not satisfied. However, it has the product–density property

(17) $$KL(P^{w}\left(w|\theta_{w}\right)\left|\right|P^{\hat{w}}\left(w|\theta_{\hat{w}}\right))=\sum_{t}^{T}\ln KL(p_{t}^{w}\left(w_{t}|\theta_{w}\right)\left|\right|p_{t}^{\hat{w}}\left(w_{t}|\theta_{\hat{w}}\right)),$$

for $p^{w}\left(w|\theta_{w}\right)=p_{1}^{w}\left(w_{1}|\theta_{w}\right)\cdot p_{2}^{w}\left(w_{2}|\theta_{w}\right)\cdots p_{T}^{w}\left(w_{T}|\theta_{w}\right)$, and $p^{\hat{w}}\left(w|\theta_{\hat{w}}\right)$ defined similarly. Hence, the MLE is an unbiased estimator of minimized Kullback–Leibler divergence:

(18) $$\begin{array}{cc} \arg\min_{\theta \in \Theta }Q_{T}\left(w_{T};\theta \right) & :=\arg\max_{\theta \in \Theta }\sum_{t=1}^{T}\ln\frac{p^{w}\left(w_{t}|\theta_{w}\right)}{p^{\hat{w}}\left(w_{t}|\theta_{\hat{w}}\right)}\\ & \;=\arg\min_{\theta \in \Theta }KL\left(P^{w}\left(w|\theta_{w}\right)\left|\right|P^{\hat{w}}\left(w|\theta_{\hat{w}}\right)\right). \end{array}$$

Note that under standard assumptions, a law of large numbers can be applied to obtain the convergence, hence, by maximizing log likelihood, we minimize Kullback–Leibler divergence. Now, we need to either find a continuously scaling function, *r*, to ensure that it also minimizes distance between the *true* measure and the modeled measure so that we may reach zero at $d_{P^{\hat{w}}}^{*}=r\;○\;d_{P}\left(P^{w},P_{\theta }^{\hat{w}}\right)∼0$. Alternatively, we find the distance metric directly. We argued above that Kullback–Leibler divergence is not a proper distance (in particular, it is not symmetric and does not satisfy the triangle inequality). However, notably useful is specifying $d_{P^{\hat{w}}}^{*}$ directly as the Hellinger distance between a modeled probability measure and the true probability measure [55]:

(19) $$H\left(P^{w}\left(w|\theta_{w}\right),P^{\hat{w}}\left(w|\theta_{\hat{w}}\right)\right)=\sqrt{\frac{1}{2}\int {\left(\sqrt{p^{w}\left(w|\theta_{w}\right)}-\sqrt{p^{\hat{w}}\left(w|\theta_{\hat{w}}\right)}\right)}^{2}dw}.$$

Specifically, the squared Hellinger distance provides a lower bound for the Kullback–Leibler divergence. Therefore, maximizing log likelihood implies minimizing Kullback–Leibler divergence, which implies minimizing the Hellinger distance. This is easily seen by the following:

**Proposition** **3.** *The squared Hellinger distance provides a lower bound to Kullback–Leibler divergence:*

$${\left(H\left(P^{w}\left(w|\theta_{w}\right)\left|\right|P^{\hat{w}}\left(w|\theta_{\hat{w}}\right)\right)\right)}^{2}\leq KL\left(P^{w}\left(w|\theta_{w}\right)\left|\right|P^{\hat{w}}\left(w|\theta_{\hat{w}}\right)\right).$$

Remark 4 below highlights that these notions do not just apply to the standard real-valued time series settings considered by Granger, but can apply to the explicit probability modeling of binary outcomes as well. Remark 4 further clarifies a result that has so far only been presented implicitly—that the probabilistic truth identified at the discussed zero-distance point may allow for a base level of entropy to exist even when all functional relationships in the process have been accounted for in a model.

**Remark** **4.** *While the paper has implicitly alluded to modeling continuous real-valued processes though the notational conventions, the connections between true probability and modeled probability are also easily made by focusing on an explicit binary outcome problem. Define cross-entropy for two discrete probability distributions p and q with the same support X:*

$$H\left(p,q\right)=E_{p}\left[-\ln q\right]=H\left(p\right)+D_{KL}\left(p||q\right)=-\sum_{x\in X}p\left(x\right)\ln q\left(x\right),$$

*in which $D_{KL}$ is Kullback–Leibler divergence, or the relative entropy of q with respect to p, and H(p) is the entropy of p. Now if p∈{y,1−y} and $q\in \{\hat{y},1-\hat{y}\}$, we can rewrite cross-entropy:*

$$H\left(p,q\right)=-\sum_{x\in X}p_{x}\ln q_{x}=-y\ln\hat{y}-\left(1-y\right)\ln\left(1-\hat{y}\right),$$

*or, for predictions generated under a set of parameters θ and a predictor x, as*

$$H\left(y,x;\theta \right)=-\sum_{t=1}^{T}y_{t}\ln p_{\theta }\left(y|x_{t-1}\right)-\left(1-y_{t}\right)\ln\left(1-p_{\theta }\left(y|x_{t-1}\right)\right).$$

*Remember that the maximum likelihood estimator maximizes the likelihood of the data under some probabilistic model. The correct likelihood in the case of binary classification is Bernoulli:*

$$p\left(y|\pi \right)=\Pi_{t=1}^{T}\pi_{t}^{y_{t}}{\left(1-\pi_{t}\right)}^{1-y_{t}},$$

*which results in the likelihood function*

$$p\left(y|x;\theta \right)=\Pi_{t=1}^{T}p_{\theta }{\left(y|x_{t-1}\right)}^{y_{t}}{\left(1-p_{\theta }\left(y|x_{t-1}\right)\right)}^{1-y_{t}}.$$

*Taking logs then gives the following log likelihood function*

$$L\left(\theta ;x,y\right)=\sum_{t=1}^{T}y_{t}\ln p_{\theta }\left(y|x_{t-1}\right)+\left(1-y_{t}\right)\ln\left(1-p_{\theta }\left(y|x_{t-1}\right)\right).$$

*This shows that negative log likelihood is proportional to Kullback–Leibler divergence and differs by the basic entropy in the data, which is constant. Maximizing the likelihood of a binary model can, thus, be understood as minimizing statistical distance toward a true probability measure; the minimum value is determined by the entropy in the observed data.*

## 5. Application

### 5.1. Practical Considerations

We continue this section first with some notes on practical considerations. Let $L_{T}\left(\theta \right)$ denote the sample log likelihood at θ∈Θ. Naturally, if $\Theta_{s}\subset \Theta$, it follows that $P_{\Theta }^{\hat{w}}⊃P_{\Theta_{s}}^{\hat{w}}$. In the limit, this means that maximizing likelihood minimizes Hellinger distance over both $P_{\Theta }^{\hat{w}}$ and $P_{\Theta_{s}}^{\hat{w}}$. Following Corollary 1, if $\theta \in \Theta_{s}$, this results in selecting $P^{\hat{w}}$ at a point in $P_{\Theta_{s}}^{\hat{w}}$ that attains equivalence to $P^{w}$. In practice, when finite data is used, two different points, one in $P_{\Theta }^{\hat{w}}\backslash P_{\Theta_{s}}^{\hat{w}}$ and one in $P_{\Theta_{s}}^{\hat{w}}$, may be obtained because the finite sample log likelihoods $L_{T}\left({\hat{\theta_{s}}}_{T}\right)$ and $L_{T}\left({\hat{\theta }}_{T}\right)$ that are available are both asymptotically biased estimators of the expected log likelihood $EL_{T}\left(\theta_{0}\right)$. This is easily shown by using a quadratic expansion [20,40]

(20) $$\lim_{T\to \infty }E\left(L_{T}\left({\hat{\theta }}_{T}\right)-EL_{T}\left(\theta_{0}\right)\right)=\lim_{T\to \infty }E\sqrt{T}{\left({\hat{\theta }}_{T}-\theta_{0}\right)}^{\prime }\frac{1}{T}L_{T}^{\prime \prime }\left(\theta_{T}\right)\sqrt{T}\left({\hat{\theta }}_{T}-\theta_{0}\right)\neq 0.$$

Under considerably restrictive conditions, the original work by [56,57] showed that the right hand-side approaches the dimension of ${\hat{\theta }}_{T}$ and, hence, an asymptotically unbiased estimator of $E\ell_{t}\left(\theta_{0}\right)$ is given by $\frac{1}{T}\sum_{t=2}^{T}\ell_{t}\left({\hat{\theta }}_{T}\right)-k$. Akaike also proposed the well-known AIC given by AIC $=2T\left(k-\frac{1}{T}\sum_{t=2}^{T}\ell_{t}\left({\hat{\theta }}_{T}\right)\right)$. Several authors have shown that the AIC can be used to consistently rank models according to Kullback–Leibler divergence in considerably more general settings, including the mis-specified case and have suggested further finite sample improvements [58,59,60]. The AIC is also valid to decide between economic theories for which no test statistics can be found [27]. This highlights that, while maximizing log likelihood over Θ is not the same objective as minimizing Kullback–Leibler divergence in finite samples, working with a complexity-penalized log likelihood (i.e., minimizing the AIC) does select the model that attains the lowest KL-bound of all considered models generated under Θ. Hence, in practice, a researcher can minimize the AIC as the practical objective to minimize Hellinger distance, and use specification tests to diagnose which of Corollaries 1 and 2 is more relevant. Since in-sample fits typically overfit data, a form of regularization would usually allow better out-of-sample results; see, for instance the (supplementary) discussion of [61] or the work of [62,63].

The challenge remains, however, that the AIC cannot be computed for all models as the degrees of freedom used in the correction is generally not a well-defined quantity for non-parametric models. As opposed to relying on in-sample corrections, cross-validation may instead be used to obtain unbiased estimates of $E\ell_{t}\left(\theta_{0}\right)$ in a setting that is more attuned to machine learning approaches, see for example [64]. Tests have been developed by [20,40,65] by following the general strategy of [66] adapted to the log likelihood case. The work has shown that choosing the model with the highest out-of-sample log likelihood equals choosing the model configuration that has achieves the highest probability of being the model that has lower Kullback–Leibler divergence. As the training *T* and validation data $\tilde{T}$ grows $T,\tilde{T}\to \infty$, this strategy chooses the model that has achieved the lowest Kullback–Leibler divergence, with probability converging to one.

### 5.2. Application to Treasury Yield Spreads and Bitcoin Spreads

The developed theory is now put into practice using daily data from short-term and long-term Treasury yield spreads and Bitcoin spreads. This is an interesting problem because each of these three assets has an important relation to inflation expectations. Rising inflation is also an acute problem, see [67,68].

The empirical strategy is as follows. First, standard linear Granger causality tests are performed as a benchmark. Next, non-parametric models will be fit in an effort to obtain an accurate-as-possible description of the true probability measure. The focus will be on maximizing out-of-sample log likelihood to minimize KL-divergence. Finally, Definitions 1 to 3 show that our conclusions about causality should be supported by a study of the probability measure that describes the causal effects. In particular, it must be decided whether this measure is a null-measure or produces real-valued data. This will be done by taking the best approximation of the true probability measure using the potential causal variable and the best approximation of the true probability measure without the potential causal variable, and (1) concluding whether the first achieves a lower KL-bound, and (2) testing whether the first is not stochastically equivalent to the latter. Section 5.2.1 first describes the data.

#### 5.2.1. Data

Dynamic interactions between spreads in short-term and long-term bond yields can naturally be expected to occur in the data. In the absence of any credit risk, the net value of future bond payments is a function of the return required based on the inflation expectation used to discount the cash stream. Each of the Treasury securities typically caries a different yield, depending on maturity, the ratio between short and long-term treasury yields signals how investors feel about the economy in the short versus long term. If the yields vary substantially throughout the day, the market is uncertain about its expectations. Investigating the flow of causality between long-term and short-term yields and the interactions with other variables has been the objective of a large number of studies. To name a few, refs. [69,70] investigate causality between bonds and credit default swaps, while [71,72,73,74,75] investigate how financial distress propagates throughout connected bond markets.

Proponents of Bitcoin have argued that it is an important hedge due to its predetermined finite supply. While Bitcoin, as an asset class, has only recently attracted the public attention of large institutional investors, many researchers have already analyzed the time-series behavior of Bitcoin prices. An overview of recent developments and more discussion on forecasting Bitcoin prices is by [76]. They investigate a large set of covariates that cover nearly all important classes of financial assets, except bonds. They conclude that the intra-day distribution of daily returns follows a nonlinear memory process better captured by machine learning methods than conventional econometric models, which is further supported by a large body of literature that has documented related modeling exorcises [77,78,79,80,81,82,83].

If investors treat Bitcoin as an inflation hedge, then the spreads may causally interact with the U.S. yield spreads. Moreover, spreads in U.S. Treasury yields will arise predominantly from uncertainty in the expectations about the U.S. economy. Bitcoin, on the other hand, as a global asset that can be exchanged peer-to-peer by individuals without the need of a financial intermediary, might react to economic uncertainty in non-U.S. economies that may have the potential to spill over. Bitcoin also trades 24 h a day, every day of the year, and so may react to turmoil that happens outside U.S. trading hours and pass it on when the markets open. At the same time, Bitcoin is a relatively small market and the large institutional investors that dominate the bond market may not be active in the Bitcoin market. Causality from Bitcoin to the bond market could, then, be unlikely. Similarly, since Bitcoin trades non-stop, information assimilates rapidly, and so it may be likely that there is no causal influence of bond spreads at the daily time frame. The different hypotheses about the causal flows will be tested first using standard Granger causality tests.

#### 5.2.2. Estimation Results

The following general system will be considered.

(21) $$\begin{array}{c} s\left(T_{t}\right)=f^{1}\left(L\left(T_{t},Q_{t},B_{t},S_{t}\right)\right)\\ s\left(Q_{t}\right)=f^{2}\left(L\left(T_{t},Q_{t},B_{t},S_{t}\right)\right)\\ s\left(B_{t}\right)=f^{3}\left(L\left(T_{t},Q_{t},B_{t},S_{t}\right)\right) \end{array}$$

In which *L* is a lag operator, *s* is a function that calculates the spread between daily highs ($h_{t})$ and lows ($l_{t}$) as the log difference $o(\log\left(1+h_{t}\right)-\log\left(1+l_{t}\right))$ where 1 is added to account for negative rates. The function *o* is a simple outlier replacement function that replaces the largest observed spread (the Corona-crash) with the second largest value. The matrices $T_{t}$, $Q_{t}$, $B_{t}$ are, respectively, the daily data of the ten-year bond, Quarterly bond, and Bitcoin price at time *t*, and $S_{t}$ is SP500 price data used as a control. The data used in the analysis runs from 1 January 2017 to 20 December 2021 and were obtained from Yahoo finance using ticker symbols ⌃TNX, ⌃IRX and BTC-USD and ⌃GSPC.

First, a linear VAR model is considered with lags selected using the AIC. All of the maximums of 10 considered lags were selected, and stability was confirmed by verifying that the largest eigenvalue of the companion matrix remained below 1 (The largest eigenvalue was approximately 0.95, indicating that the process was stable but strongly dependent. Results were also generated using differenced data, which resulted in stronger causal linkages. Results are implemented in the code available with the paper but not shown here for compactness. see Supplementary Materials). Conditional Granger tests for causality are calculated by applying an F-test to the squared residuals of the model with and without the lags of a variable of interest in the presence of the autoregressive lags and the other control variables. The table below reports the *p*-values.

There are two important results in Table 1. First, the AIC, as an in-sample estimator of KL-divergence, selects a very large number of lags. The BIC is not an estimator of KL-divergence, see [84], but is a closely related Bayesian alternative to the AIC that is widely used. It places a larger penalty on the number of parameters and, as such, behaves somewhat similar to the corrected AIC in finite samples. The table shows that with this alternative criterion, a vastly different model is chosen. As Equation (20) showed, and the discussion after mentioned, the in-sample estimator of log likelihood is a biased estimator of expected log likelihood and, in practice, it is difficult to determine the appropriate penalty. In Table 1, two vastly different results are obtained. In both cases, however, the *p*-values of all causality tests are small. Both models suggest that there are strong causal linkages between spreads in all three markets. The statistical significance is somewhat dubious: the VAR(AIC) suggests that the causal flow of financial distress spills over in all directions. Moreover, Table 1 shows that, by adding more lags the significance of the causality tests increases, while it is likely that with 10 lags the model is trying to approximate a nonlinear process and the extremely high number of parameters involved in this approximation are likely over-fitting the data.

The section will now use an RF model to better approximate $\left(f^{1},f^{2},f^{3}\right)$. The implementation used is that of [85], all possible tuning parameters are considered. The consistency of the RF in a time-series context under the assumption of data generated by a nonlinear autoregressive process is developed by [86]. As the previous sections detailed, the out-of-sample estimate of log likelihood is proportional to KL-divergence but RF models are typically not estimated using an in-sample log likelihood approach. A log likelihood function can nevertheless still be specified for out-of-sample predictions. To retain simplicity of the example, the commonly used Gaussian formulation is used:

(22) $$\ell \left(v_{t},\mu_{t},\sigma_{t}\right)=\sum_{t}^{T}\frac{1}{2}\left(2\pi \sigma_{t}^{2}\right)-\frac{{\left(v_{t}-\mu_{t}\right)}^{2}}{2\sigma_{t}^{2}}$$

In this function, $v_{t}$ are holdout validation samples at time *t* and $\mu_{t}$ is the mean parameter, which will be substituted by the conditional means predicted on the holdout data by the model. Note that $\sigma_{t}$, the variance parameter, is allowed to be time-varying. This is important because spread data is not homoskedastic, and the variance varies over the time dimension [87,88]. The log likelihood function thus allows for heteroskedasticity, the standard literature is followed and $\sigma_{t}$ estimated using an ARMA-GARCH model. (The algorithm is as follows. Consider the time-varying density $F_{t}=\left(\mu_{t},\sigma_{t},\vartheta \right)$, where $\mu_{t}$ is a conditional mean process. For simplicity, it is defined as an ARMA (1,1) process

(23) $$\mu_{t}=c+\phi \mu_{t-1}+\theta \varepsilon_{t-1}+\varepsilon_{t},$$

and the conditional variance, again for simplicity, is specified as a GARCH process of order (1,1):

(24) $$\sigma_{t}^{2}=\omega +\alpha \varepsilon_{t-1}^{2}+\beta \sigma_{t-1}^{2}$$

with $\sigma_{t}^{2}$ as the conditional variance, ω an intercept, and *L* the back-shift operator. The vector ϑ specifies any remaining parameters of the distribution, in this case, the log likelihood is estimated using the Gaussian distribution in line with the validation criterion).

The RF models use three lags of the spread data so that the BIC-selected VAR model is nested. Several other features are added that may help describe the long-term dependencies captured by the AIC-selected model more accurately. In particular, a relative strength index (RSI) of all close values, including the SP500 close, is calculated. This is a standard indicator on [0,100], described in many resources that compare average upward movement to average downward movements over a look-back period. The standard period of 14 days is used along with a look-back of 14 weeks. The latter is also calculated using the spread data. This way, the model may learn different dependencies in periods of sustained decline, increase, or stability, in spreads and prices. The bootstrap sampling algorithm of the RF allows for case weights, effectively increasing the probability that highly weighted cases are over-represented in the random base learners, see [85]. This is exploited; $\sigma_{t}^{2}$ is standardized in the training data to be used as case-weights so that observations during more volatile periods feature more frequently in the sampling scheme.

The out-of-sample log likelihood is cross-validated using Equation (23), using 20 folds so that each validation sample has approximately 60 observations. The splits are generated using a stratified sampling approach that conditions on the RSI of the SP500. In other words, validation samples are chosen so that each validation sample equally represents days of under-bought, over-bought, and neutral stock market territories. The split is generated once and kept identical for each model so that the results can be directly compared. In total, an out-of-sample log likelihood value is generated for each observation so the sum of the log likelihood is taken to obtain an estimate of total out-of-sample log likelihood.

The results in Table 2 show the following. First, the nonlinear autoregressive models (indicated by the rows that apply a lag operator to the dependent variable listed in each column) all out-compete the VAR model that used all variables. According to the theory of the paper, the causal results obtained using the linear Granger causality tests in Table 1 should thus be discarded in favor of the theory that each variable follows a nonlinear autoregressive process that only makes possible reference to the SP500 but not the other variables of interest. For instance, the VAR of the ten-year Treasury yield spreads reach an out-of-sample log likelihood of 3916.77, while the nonlinear RF model reached a log likelihood of 3932.78 without using the lagged quarterly yield spreads or Bitcoin spreads. The differences in log likelihood are even larger for the models for quarterly Treasuries spreads and Bitcoin spreads.

Table 2 contains only evidence for two possible causal linkages. First, the model for the spreads on the ten-year that reached the lowest KL-bound used the lags of the quarterly yield data. This suggests that causality, in financial distress, may run from the short-term bonds to the long-term bonds. This is sensible; acute economic fears may impact short-term expectations more heavily, and the reaction in the short-term yields may trigger further fears about longer-term economic expectations. The second causal link could run from the Bitcoin market to the quarterly bonds. This is not far-fetched: Bitcoin trades non-stop and so any event globally can impact the Bitcoin market immediately, whereupon the increased fear in the Bitcoin market could then trigger further reactions in the short-term bond market, which would be more susceptible to short-term economic fears. However, the point increase in log likelihood that backs this hypothesis is small compared to the model that only used endogenous lags and control data.

Recall Remark 1: to test whether the evidence for causality is strong enough; it is important to test whether the probability measures that achieved the lowest KL-bound are stochastically different from those that exclude the causal linkages. A Kolmogorov–Smirnov test, under the null of distributional equivalence against a two-sided alternative, is computed. For the ten-year yield spread model, the *p*-value is 0, so the null is overwhelmingly rejected. The analysis, thus, concludes that the best possible hypothesis is that disruptions in the short-term bond market cause further disruption in the longer-term bond market. The test for distributional equivalence between the model with and without Bitcoin data has a *p*-value of 0.8591. In other words, the null of equivalence cannot be rejected and, while the model that used Bitcoin data reached the lowest KL-bound, the analysis does not find significant evidence for a causal flow from the Bitcoin market to the short-term Treasuries as the modeled probability measure is not significantly distinguishable from the competing non-causal measure. This suggests that the probability measure that describes the causal effects in Definition 2 is not distinguishable from that of Definition 1, and so Corollary 1 or 2 remain inconclusive. The final conclusion that causal flows are thus parsimonious is far more likely than the result obtained with the VAR, which suggested that causality flows significantly in all directions.

## 6. Concluding Remarks

This paper has developed a probabilistic theory of causation using measure-theoretical concepts. It discussed how probabilistic truths can be approximated by minimizing distance to the true probability measure over a space of measures in which each element is associated with a probabilistic theory about causation. This notion is flexible and has allowed for a wide range of models to be used for causal inference, including linear and nonlinear dynamical models. The theory has been applied using daily data on yield spreads to test how uncertainty around short-term and long-term expectations about future inflation interact with uncertainty in the daily Bitcoin price. The results were contrasted with those obtained using standard linear Granger causality tests. While linear Granger causality relies on models that assume a constant causal influence from one variable onto another, specified by static parameters, the analysis has shown that time-varying properties of the auto-regressive process provides a better description of the data. While the linear Granger causality tests finds significant causal influence in all directions, the suggested measure-theoretic approach to causality testing, using, in this example, a random forest model, found only one significant causal link that ran from financial distress in the short-term bond market to uncertainty in the long-term bond market.

As with Granger’s approach, a convincing theory of how causes produce effect is not necessarily a prerequisite to making correct causal inferences. Clear hypotheses about causal relations may, however, help guide the inference by helping design better models. However, whereas Granger’s definition “is based entirely on the predictability of some series” [5], the ideas of the current paper start with the notion that true probabilistic laws exist and can, and should, correctly be approximated to infer causal structures from data. A conclusion from this is that researchers interested in causal analysis should aim to develop strong out-of-sample predictions, as Granger’s techniques applied to inaccurate models may provide an overly enthusiastic description of causal linkages.

The general ideas of the paper differ from the linear Granger tests in terms of result, but share a similarity in thought process. Granger’s statement about causality followed from the premises that causes occur before effects and that causes contain unique information about their effect, and so that any causal variable must help forecast outcomes after other variables have been used first. For this reason, many refer to Granger causality as predictability. This paper defined causality directly in terms of the probability measures that define a stochastic process. This, in turn, places the emphasis on finding the best approximation of that probability measure. The theory developed here shows that minimizing KL-divergence implies minimizing distance between a model and the true probability measure and shows that maximizing out-of-sample log likelihood implies minimizing KL-divergence. This does not require parametric models or the degrees of freedom to be known. Instead, the KL-ranking of competing models can be directly read from the out-of-sample log likelihood. The stochastic equivalence, or difference, between probability measures that are induced by causal flows, or from autoregressive properties only, can subsequently be tested. The theory provides practitioners guidance for developing causal models using new machine learning methods that have, so far, remained relatively underutilized in this context.

## Figures and Tables

**Table 1:** *p*-values for Granger causality tests using VAR methods. Columns indicate the dependent variables, rows correspond to exogenous lags tested for causality. Each linkage is tested in the presence of lagged SP500 spreads as a control. Note that the BIC is not an estimator of KL-divergence, but it is widely used as a Bayesian alternative that places a higher penalty on dimensionality. Blank entries are intentionally left so, as they refer to endogenous linkages.

		AIC (lags = 10)			BIC (lags = 3)	
	$s(T_{t})$	$s(Q_{t})$	$s(B_{t})$	$s(T_{t})$	$s(Q_{t})$	$s(B_{t})$
$L(s\left(T_{t}\right))$		0	0.0225		0	0.2207
$L(s\left(Q_{t}\right))$	0		0.0021	0.0183		0.0450
$L(s\left(B_{t}\right))$	0.0142	0.0083		0.1093	0.0635	

**Table 2:** Cross-validated log likelihood for different models. Columns indicate the dependent variables, rows correspond to exogenous lagged data that are used by the models in addition to the control data. For each dependent variable, the model that achieved the lowest KL-divergence is marked by *.

	$s(T_{t})$	$s(Q_{t})$	$s(B_{t})$
VAR	3916.77	4084.68	2204.91
RF			
All	3989.14	4239.42	2251.06
$L(s\left(T_{t}\right))$	3932.78	4230.44	2251.68
$L(s\left(Q_{t}\right))$	3991.24 *	4240.54	2251.46
$L(s\left(B_{t}\right))$	3932.90	4242.67 *	2251.84 *

## Data Availability

Code to reproduce the analysis is available along with the paper or can alternatively be requested from the author. The data is taken from Yahoo, the code calls the data from their API.

## Supplementary Materials

The following supporting information can be downloaded at: https://www.mdpi.com/article/10.3390/e24010092/s1.

## Abbreviations

The following abbreviations are used in this manuscript:

DGP	data-generating process
MLE	maximum likelihood estimator
IRF	impulse response function
VAR	vector auto-regression
RF	random forest
RSI	relative strength index

## Appendix A. Proofs

### Appendix A.1. Proof for Proposition 1

**Proof.** By construction of the criterion, as stated in Assumption 1, $\arg\min_{\theta \in \Theta }$$Q_{\infty }\left(\theta \right)$ is its minimizer, and, by assuming $\theta_{0}\in \Theta$, it is also equal to $\theta_{0}$. Hence, item 2 is equivalent to item 1 by definition under correct specification. The equivalence of the deterministic limit criterion (item 2) as a function describing the divergence of the underlying probability measures of w and $\hat{w}$ (item 4) is assumed, however, given a limit criterion function $Q_{\infty }:\Theta \to R$ and a flexible definition of divergence (e.g., a pre-metric, such as the KL-divergence), it is often possible to find a divergence $d_{P}:P_{\Theta }\times P_{\Theta }\to R_{\geq 0}$ on the space of probability measures satisfying $\arg\min_{\theta \in \Theta }d_{P}\left(P^{w},P_{\theta }^{\hat{w}}\right)=\arg\min_{\theta \in \Theta }Q_{\infty }\left(\theta \right)$. The KL-divergence example is provided in this paper in the context of the maximum likelihood criterion. By the assumption that *r* exists, the deterministic limit criterion that minimizes divergence, is also the minimizer of a distance metric $d_{P}^{*}\left(P^{w},P_{\theta }^{\hat{w}}\right)$, hence item 4 is also equivalent to item 2. Finally, since $f_{W}:\Theta \to F_{\Theta }\left(W\right)$ is injective, $\left(P^{w},P_{\theta }^{\hat{w}}\right)\equiv d_{F}^{*}\left(f^{w},f\left(\cdot ,\theta \right)\right)\;\forall \;\theta \in \Theta$ and $d_{F}^{*}$ is a metric on $F_{\Theta }\left(W\right)$, $\theta_{0}$ is also the minimizer of $d_{F}^{*}\left(f^{w},f\left(\cdot ,\theta \right)\right)\;\forall \;\theta \in \Theta$ so that item 3 is equivalent to item 2. □

### Appendix A.2. Proof for Proposition 2

**Proof.** The result follows immediately by the arguments used in proposition 1 dropping only the first equivalence. □

### Appendix A.3. Proof for Proposition 3

**Proof.** First, Hellinger distance is

$$H\left(P^{w}\left(w|\theta_{w}\right),P^{\hat{w}}\left(w|\theta_{\hat{w}}\right)\right)=\sqrt{\frac{1}{2}\int {\left(\sqrt{p^{w}\left(w|\theta_{w}\right)}-\sqrt{p^{\hat{w}}\left(w|\theta_{\hat{w}}\right)}\right)}^{2}dw},$$

hence,

$${\left(H(P^{w}\left(w|\theta_{w}\right),P^{\hat{w}}\left(w|\theta_{\hat{w}}\right))\right)}^{2}=\frac{1}{2}\int {\left(\sqrt{p^{w}\left(w|\theta_{w}\right)}-\sqrt{p^{\hat{w}}\left(w|\theta_{\hat{w}}\right)}\right)}^{2}dw.$$

Now, the R.H.S. can be written as

$$\frac{1}{2}\int p^{w}\left(w|\theta_{w}\right)dw+\frac{1}{2}\int p^{\hat{w}}\left(w|\theta_{\hat{w}}\right)dw-\int \sqrt{p^{w}\left(w|\theta_{w}\right)p^{\hat{w}}\left(w|\theta_{\hat{w}}\right)}dw.$$

The integral of a probability density over its domain equals 1, hence the sum of the first two terms is 1, hence this can be rewritten as

$$1-\int \sqrt{p^{w}\left(w|\theta_{w}\right)p^{\hat{w}}\left(w|\theta_{\hat{w}}\right)}dw.$$

This has an upper bound, provided by the inequality

$$1-\int \sqrt{p^{w}\left(w|\theta_{w}\right)p^{\hat{w}}\left(w|\theta_{\hat{w}}\right)}dw\leq -\ln\int \sqrt{p^{w}\left(w|\theta_{w}\right)p^{\hat{w}}\left(w|\theta_{\hat{w}}\right)}dw.$$

Write R.H.S. as $-\ln\int \left[\sqrt{\frac{p^{\hat{w}}\left(w|\theta_{\hat{w}}\right)}{p^{w}\left(w|\theta_{w}\right)}}p^{w}\left(w|\theta_{w}\right)\right]dw$ and to obtain the upper bound

$$-\ln\int \left[\sqrt{\frac{p^{\hat{w}}\left(w|\theta_{\hat{w}}\right)}{p^{w}\left(w|\theta_{w}\right)}}p^{w}\left(w|\theta_{w}\right)\right]dw\leq -\int \left[\ln\sqrt{\frac{p^{\hat{w}}\left(w|\theta_{\hat{w}}\right)}{p^{w}\left(w|\theta_{w}\right)}}p^{w}\left(w|\theta_{w}\right)\right]dw,$$

by applying Jensen’s inequality, which can be applied to the integral case, since any random variable whose distribution admits a probability density function has the expected value represented by the integral over the full range of the density. Finally, define the R.H.S. as

$$E\int \left[\ln\frac{p^{w}\left(w|\theta_{w}\right)}{p^{\hat{w}}\left(w|\theta_{\hat{w}}\right)}p^{w}\left(w|\theta_{w}\right)\right]dw=-\int \left[\ln\sqrt{\frac{p^{\hat{w}}\left(w|\theta_{\hat{w}}\right)}{p^{w}\left(w|\theta_{w}\right)}}p^{w}\left(w|\theta_{w}\right)\right]dw,$$

and conclude that the last expression is equivalent to the Kullback–Leibler divergence by an elementary row operation.

$$E\int \left[\ln\frac{p^{w}\left(w|\theta_{w}\right)}{p^{\hat{w}}\left(w|\theta_{\hat{w}}\right)}p^{w}\left(w|\theta_{w}\right)\right]dw\equiv KL\left(P^{w}\left(w|\theta_{w}\right)\left|\right|P^{\hat{w}}\left(w|\theta_{\hat{w}}\right)\right).$$

□
